# Cryptogenic Cirrhosis and Hepatopulmonary Syndrome in a Boy with Hepatic Hemangioma in Botswana: A Case Report and Review of the Literature

**DOI:** 10.1155/2017/7940365

**Published:** 2017-12-19

**Authors:** Francis Msume Banda, Jeremy S. Slone, Alan Anderson, Marisa Beretta, Priya Walabh, Jerome Loveland, Simon Nayler, Farirai Fani Takawira

**Affiliations:** ^1^Department of Paediatrics and Adolescent Health, Faculty of Medicine, University of Botswana, Gaborone, Botswana; ^2^Baylor College of Medicine, Texas Children's Cancer and Hematology Centers, Houston, TX, USA; ^3^Paediatric Liver Transplant Unit, Wits University Donald Gordon Medical Centre, Charlotte Maxeke Johannesburg Academic Hospital, Johannesburg, South Africa; ^4^Department of Paediatric Surgery, School of Clinical Medicine, University of Witwatersrand, Johannesburg, South Africa; ^5^Wits University Donald Gordon Medical Centre, Drs Gritzman & Thatcher Inc., Johannesburg, South Africa; ^6^Department of Paediatrics, Steve Biko Academic Hospital, University of Pretoria, Pretoria, South Africa

## Abstract

Hepatic hemangiomas are considered to be the most common benign tumors of the liver. They are often found incidentally while investigating for other causes of liver disease. Hemangiomas that are less than 10 cm are not expected to cause any problems. Typically, they do not enlarge and, apart from regular follow-up, no definitive treatment is indicated. This is a posthumous case report of a male child with a medium-sized hemangioma from infancy, complicated by cryptogenic cirrhosis and hepatopulmonary syndrome. It demonstrates the challenges of managing a child with such complicated conditions in a resource-limited setting.

## 1. Case Report

An 8-month-old boy was admitted to the Paediatric Ward of Princess Marina Hospital (PMH), the main government referral hospital in Botswana, with abdominal distention and nonbile-stained, nonprojectile vomiting for 2 weeks. No fever, irritability, diarrhoea, or poor feeding was reported. The child was born at term by uncomplicated spontaneous vaginal delivery to a mother who was already on highly active antiretroviral therapy (HAART) before pregnancy. His birth weight was 2950 g. The patient was enrolled into the Perinatal Mother-to-Child Transmission programme as per Botswana guidelines [1]. His neonatal period was uneventful, and his HIV-DNA polymerase chain reaction was negative at 18 months of age.

The child was previously admitted to PMH when he was 3 months old with culture-negative sepsis and received empiric intravenous antibiotics. On examination during an admission at the age of 8 months, he was slightly lethargic with dry mucus membranes and a capillary refill time of less than 2 seconds. The anterior fontanelle was slightly sunken. Oxygen saturation in room air was 99%. Vital signs and anthropometric measurements were within normal limits. There was no respiratory distress or dysmorphic features. In addition, the patient had mild scleral icterus. The abdomen was distended and soft; however, an irregular and nontender hepatomegaly of 6 cm was palpable below the right subcostal margin, with no splenomegaly. The rest of the physical examination was unremarkable.

Full blood count showed mild leukocytosis and normocytic anemia, whilst liver function tests demonstrated a mild transaminitis. Renal function tests showed raised creatinine and urea consistent with dehydration. Serum electrolytes and thyroid function tests were normal. Hepatitis virus serology was negative (Table 1). Urine and blood cultures were negative after 5 days of incubation. Chest X-ray was unremarkable.

Abdominal ultrasound showed hepatomegaly (5 cm × 5 cm × 5 cm) with mild ascites. The spleen, gallbladder, pancreas, kidneys, aorta, and inferior vena cava were normal. There was no retroperitoneal lymphadenopathy. Magnetic resonance imaging scan of the abdomen 3 months later showed mild hepatomegaly with a large well-encapsulated exophytic lesion (5 cm × 5 cm × 5.3 cm) in the left lobe of the liver consistent with hemangioma. He was started on propranolol therapy to treat the hemangioma. Between the age of 1 and 4 years, he had several abdominal ultrasound scans, and all showed that the liver mass remained the same size.

**Figure 1:**
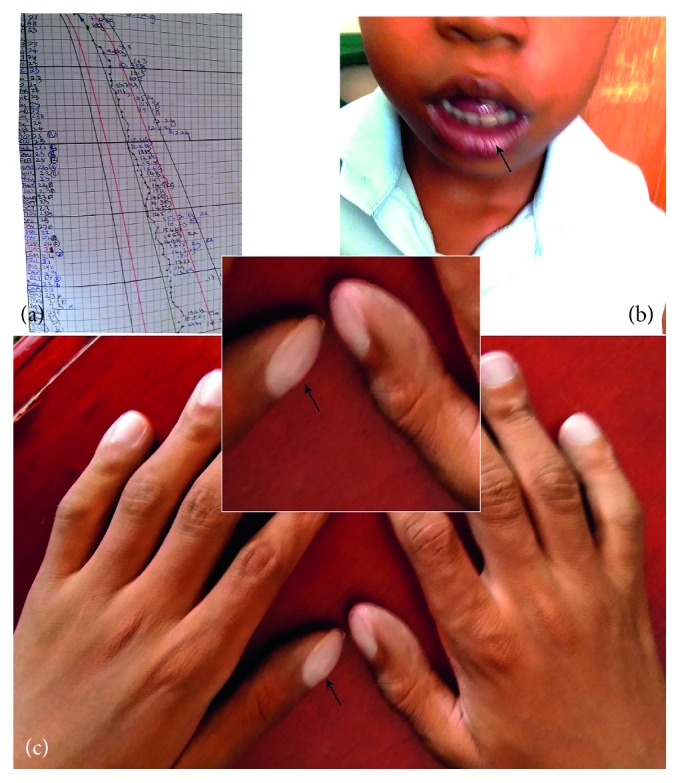
(a) Weight-for-age plot for the patient. Growth consistently above 0 Z-score (middle line on the image) throughout the under-five period. (b) Central cyanosis (arrow). (c) Finger clubbing (arrows).

At the age of 4 years, he was growing well, with weight plotting consistently above 0 Z-score weight-for-age. Developmental milestones were appropriate for age. However, for the first time, he was noted to be cyanosed, both peripherally and centrally, with oxygen saturation of 70% in room air, improving to 85% with supplemental oxygen by nasal prongs. He had developed conjunctival chemosis and finger clubbing (Figure 1). He had no jaundice, pallor, oedema, or lymphadenopathy. In addition, he had a hyperdynamic apex in the 5th intercostal space in the left midclavicular line. The first heart sound was normal, and there was a 2/6 ejection systolic murmur at the tricuspid area that could also be heard over the praecordium. There was no triple rhythm. In addition, the patient had mild subcostal and intercostal recessions, but the lung fields were clear on auscultation. At this point, the abdomen was not distended, but there was hepatosplenomegaly, extending 6 cm below the subcostal margin. There was no hepatic bruit or ascites.

Of note on the full blood count were polycythemia, microcytosis, and thrombocytopenia, with a hemoglobin of 17.1 g/dL, mean corpuscular volume of 67.5 fL, and platelet count of 100 × 109/L. Liver function tests showed no significant changes from previous. Coagulation profile was normal (Table 1).

**Figure 2:**
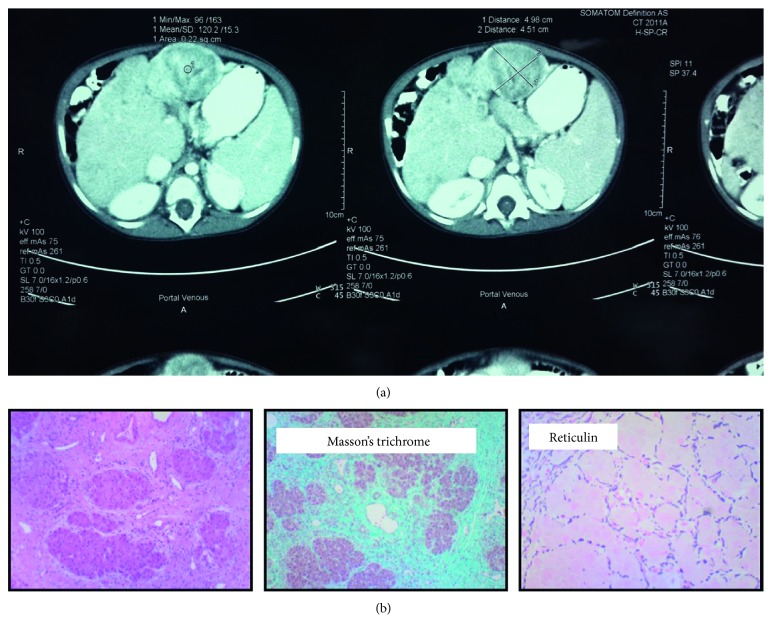
(a) CT scan of the abdomen. Note the hepatic hemangioma (crossed lines 1 and 2). (b) Liver histology. Left to right: Liver parenchyma is present which, although subcapsular, shows evidence of marked distortion of the normal architecture, with fibrosis present surrounding regenerative-type nodules. Masson trichome stain shows extensive interstitial fibrosis surrounding regenerative-type nodules, as can be appreciated on the microphotograph. Reticulin stain shows twinning of the cell plates, which can be appreciated on the microphotograph.

With these examination and laboratory findings, we strongly suspected hepatopulmonary syndrome although he had no overt clinical signs of hepatic cirrhosis. We thought that he had developed portal hypertension, which could explain the splenomegaly and the thrombocytopenia. He was referred to a hepatobiliary specialist in South Africa, where computed tomographic scan of the abdomen and pelvis showed a large mass in segment III of the left lobe of the liver (4.98 × 4.51 cm), that was suggestive of hemangioma, and had not changed in size over the years (Figure 2). There was no intrahepatic bile duct dilatation. Gallbladder, pancreas, kidneys, bowel, and mesentery were normal. There was a 10 cm splenomegaly with no ascites.

Transesophageal echocardiography showed an anatomically normal heart with normal valves, normal chamber size and function, and no intracardiac shunts (patent foramen ovale or septal defects). The internal diameters for the left ventricle were 42.7 mm (diastolic) and 28.1 mm (systolic). Ejection fraction was 64% (normal). Shortening fraction was 34% (normal). All major vessels were of normal diameter (inferior vena cava: 5.52 mm; right pulmonary artery: 8.32 mm; main pulmonary artery: 15.19 mm; aorta: 22.63 mm). There were no features of pulmonary hypertension. Contrast enhancement with saline showed a delayed opacification of the left atrium (positive bubble test), suggesting an intrapulmonary right-to-left shunt most probably due to pulmonary vascular dilatation. The PaO_2_ was <34 mmHg, and oxygen saturation in room air was 65% (80% on nasal oxygen).

Exploration with a view to resection of the liver lesion revealed no mass. Instead, advanced cirrhosis was noted.

A wedge liver biopsy showed grossly distorted architecture with regenerative micronodules of hepatocytes surrounded by dense fibrotic septa showing portoportal and portovenous linkage (Figure 2(b)(b)). Mild inflammation was present in the septa, but there was no definitive ductal plate abnormality (bile duct malformation). There were slightly dilated portal venules. The Masson trichrome stain confirmed dense well-established fibrosis in the bridging septa, and the reticulin stain confirmed twinning of the cell plates. There was no evidence of cholestasis and a range of special stains excluded Wilson's disease, alpha-1 antitrypsin disease, and metabolic diseases. No hemangioma was represented on the wedge biopsy.

All other studies, including hepatitis virus serology, autoimmune screen, and copper studies, were also negative. The patient was assessed as having cryptogenic cirrhosis and severe hepatopulmonary syndrome (in view of chronic liver disease, chronic hypoxia, and the delayed opacification saline bubble test). He was assessed in the liver transplant unit and was considered to be a candidate for liver transplantation. However, in view of the severity of the hepatopulmonary syndrome, he was put on home oxygen to try and prevent his pulmonary status from deteriorating and improve his arterial oxygen content. He was advised to return 2 months later when he would be worked up for liver transplantation. Sadly, he was unable to return to South Africa for his next appointment due to financial constraints. He was on home oxygen until his demise. An autopsy was offered, but the parents declined to have it done.

## 2. Discussion

Hepatic hemangiomas are considered the most common benign tumors of the liver. They are often found incidentally while investigating for other causes of liver disease. They are classified according to their size into capillary (less than 3 cm in greatest diameter), medium (between 3 cm and 10 cm), and giant or cavernous (more than 10 cm) [2]. Our child had a medium-sized hemangioma, for which, apart from regular follow-up, no active interventions are required [3]. The fact that it neither increased in size nor was it found on laparotomy suggests that it did not play any role in the clinical deterioration of our child and had most likely undergone spontaneous resolution.

Various treatment options for hemangiomas have been described in the literature. These include the use of drugs like corticosteroids, interferon-*α*, and vincristine, as well as surgical excision, laser ablation, embolization, and liver transplantation [4]. We chose propranolol, a *β*-adrenergic antagonist, which is an efficacious and safe treatment for hemangiomas [5, 6]. It is easy to source in our government hospital, so our patient was therefore assured of a constant supply.

Hepatopulmonary syndrome is a well-recognized pulmonary complication of liver cirrhosis. It is usually diagnosed in the presence of a triad of liver disease, oxygenation defect, and intrapulmonary microvascular dilatations [7] on pulmonary angiography. However, contrast-enhanced transthoracic echocardiography with saline (bubble test, as done in our patient) is an equally reliable investigation to ascertain the presence of pulmonary vascular dilatation. It is highly sensitive in confirming intrapulmonary vascular dilatations, such that it is regarded as the standard in diagnosing hepatopulmonary syndrome [8]. However, it does not distinguish between saline bubble passage through dilated pulmonary capillaries and arteriovenous malformations. It would have been best to confirm this with pulmonary angiography or technetium-99m MAA nuclear study [9, 10], which we were unable to do in our case.

Dyspnea has both positive and negative predictive values of 100% for hepatopulmonary syndrome. Cyanosis has a negative predictive value of 97% while finger clubbing has been reported to have a negative predictive value of up to 75% [11]. Our patient did not have spider angiomas, although they have been widely described as cutaneous markers of the hepatopulmonary syndrome, especially in the setting of intrapulmonary vascular dilatations [11, 12].

Hepatopulmonary syndrome is classified as mild (PaO_2_ ≥ 80 mmHg), moderate (PaO_2_ ≥ 60 < 80 mmHg), severe (PaO_2_ ≥ 50 < 60 mmHg) and very severe (PaO_2_ < 50 mmHg). These patients consistently have elevated serum levels of nitric oxide and carboxyhemoglobin, both of which have been postulated to play a role in the pathogenesis of the syndrome [8] and normalize after liver transplantation. The nitric oxide is linked to the causation of the pulmonary vascular dilatation [13] while the carboxyhemoglobin is linked to the severe hypoxemia [14]. However, the defect in oxygenation is largely attributed to increased blood flow through the dilated pulmonary vessels in the alveolar capillary bed. While alveolar ventilation remains constant, the increased blood flow through the low pressure system creates a ventilation-perfusion mismatch [15]. This explains why PaO_2_ increases when a patient with hepatopulmonary syndrome breathes 100% oxygen [16].

In infancy, biliary atresia and genetic metabolic disease are the commonest cause of chronic liver disease, while chronic viral hepatitis and autoimmune disease predominate after infancy. Cryptogenic cirrhosis accounts for 5–15% overall [17]. Cryptogenic cirrhosis and cirrhosis due to hepatitis B virus infection are the commonest causes of hepatopulmonary syndrome in the medical literature [11]. Our patient most likely had cryptogenic cirrhosis based on the physical findings and nonconclusive results of extensive investigations. The patient lived for an additional 2.5 years despite the severe hypoxia.

The patient had two admissions in infancy due to severe illnesses. We did not find their aetiology, and investigations did not point towards immunodeficiency. However, we are also aware that HIV-exposed uninfected infants are at increased risk for severe infections in the first year of life [18].

Liver transplantation is the only successful treatment for hepatopulmonary syndrome [19]. Patients with severe hepatopulmonary syndrome do achieve a resolution of the hypoxemia after liver transplantation [20–22], although such patients often have a very difficult posttransplant existence, characterized by many intensive care admissions, long recovery periods, and oxygen dependence, with a significant risk of mortality [23, 24]. It is generally accepted that posttransplantation mortality is directly related to the severity of the hypoxemia before the transplantation [23]. A baseline PaO_2_ ≤ 50 mmHg has been associated with poor prognosis whether transplantation is done or not [25], and is considered the strongest predictor of death [26]. A well-established posttransplant health care system and a conducive socioeconomic environment at home are other important considerations [27]. The current protocol regarding severe hepatopulmonary syndrome and liver transplantation at our referral facility requires that families be extensively counseled regarding the dramatically increased mortality and morbidity rate and extended the choice to progress to transplantation. A child with severe hepatopulmonary syndrome will not be excluded from transplantation unless he/she is excluded by factors that are applicable to all other transplant candidates. The main limiting factors to transplantation in our child were the poor socioeconomic status of the family (parents were unemployed and did not have private medical aid) and the child's inability to return for the 2 months' appointment, which cast considerable doubt on the child's readiness to access the facility after transplantation, as currently there is no paediatric posttransplant health care system in Botswana.

## 3. Conclusion

Our case report, the first of its kind to be reported from Botswana, demonstrates the difficult challenges of managing a child with cryptogenic cirrhosis and hepatopulmonary syndrome in the setting of liver transplant considerations.

## Supplementary Material



## Figures and Tables

**Table 1:** Laboratory results.

Age	8 m	4 y
FBC^a^		
WBC (5−10 × 10^3^/mL)	12	8
Neutrophils (40−60%)	68	42
Lymphocytes (20−40%)	31	28
Hb (11.5−14 g/dL)	10	17.1
MCV (75−90 fL)	88	67.5
Platelets (145−450 × 10^3^/mL)	470	100
LFTs^b^		
Total bilirubin (<17.1 *µ*mol/L)	0.8	0.53
Total protein (63−80 mg/dL)	76.3	71.7
Albumin 35−55 mg/dL	34.5	33
ALP (50−160 IU/L)	257.8	262
ALT (0−30 IU/L)	78	75.4
AST (0−40 IU/L)	86.3	84
GGT (0−30 IU/L)	63.6	69
LDH (140−280 IU/L)	220	262
U and E^c^		
Na (135−145 mmol/L)	142	—
K (3.5−5.5 mmol/L)	3.8	—
Cl (98−106 mmol/L)	101	—
Urea (7–14 mmol/L)	22	—
Creatinine (88−176 *µ*mol/L)	83.7	—
TFTs^d^		
FT4 (3.5−6.5 *µ*g/dL)	3.78	—
T3 (11.5−22.7 ng/dL)	20.13	—
TSH (0.35−5.5 *µ*U/mL)	4.81	—
Coagulation		
aPTT (30−45 s)	35	30.6
INR (0.8−1.2)	0.97	1.13
Serum serology studies		
Hepatitis A and B viruses	Negative	Negative
Autoimmune screen		
ANAs	—	Negative
Serum immunoglobulins	—	Normal

^a^Full blood count; ^b^liver function tests; ^c^urea and electrolytes; ^d^thyroid function tests.
