# Supplementary figures and images for: Cryptogenic Cirrhosis and Hepatopulmonary Syndrome in a Boy with Hepatic Hemangioma in Botswana: A Case Report and Review of the Literature
Source: Case Rep Pediatr. 2017 Dec 19;2017:7940365. doi: 10.1155/2017/7940365 (PMC5749285; doi:10.1155/2017/7940365)

**LIVER BIOPSY REPORT**


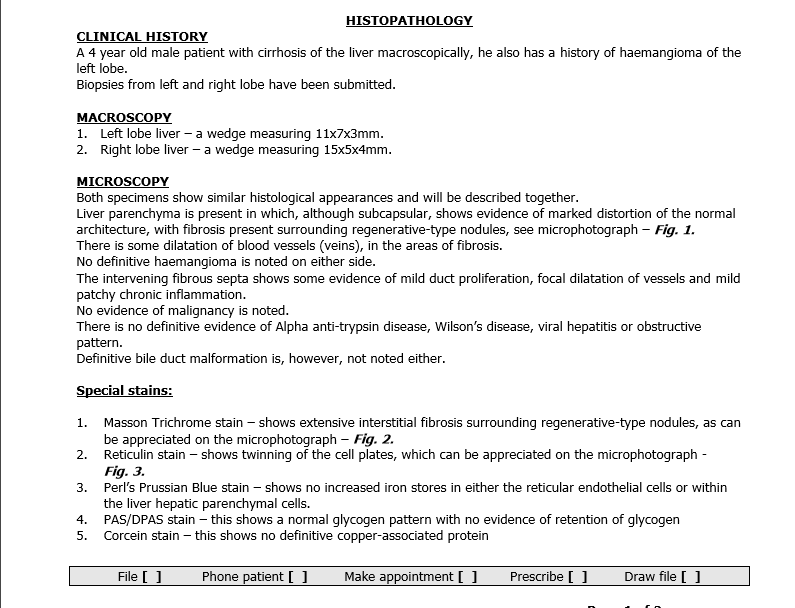


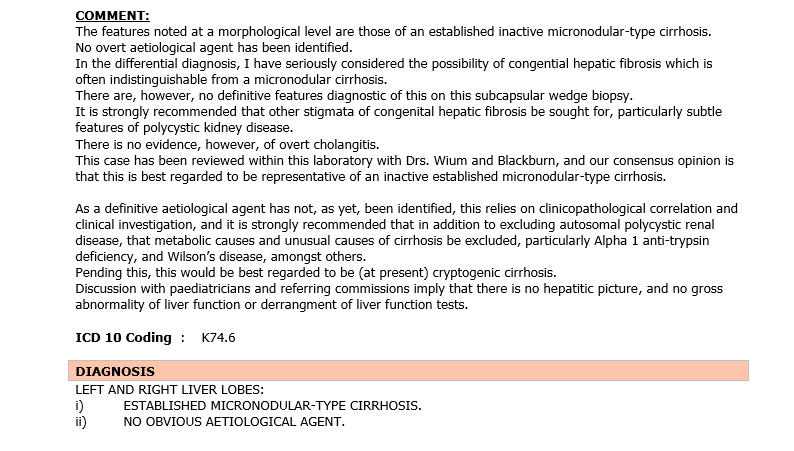


**CARDIOLOGY REPORT:**


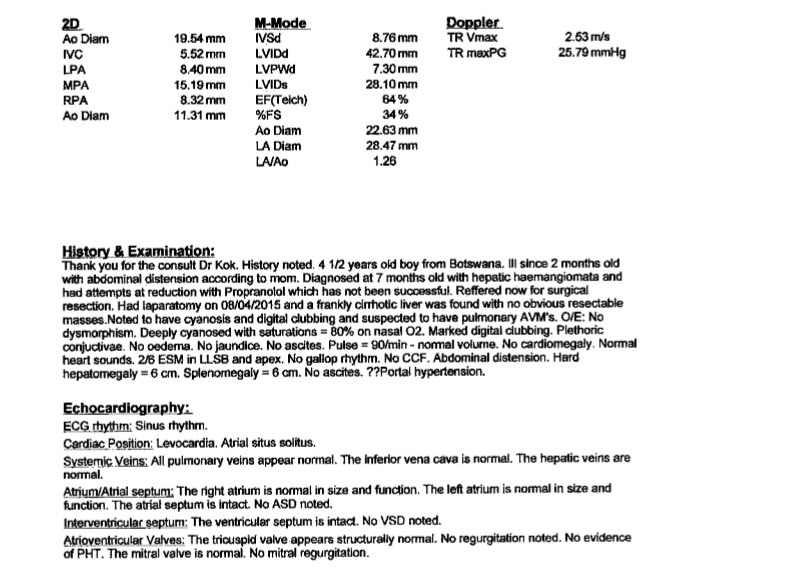


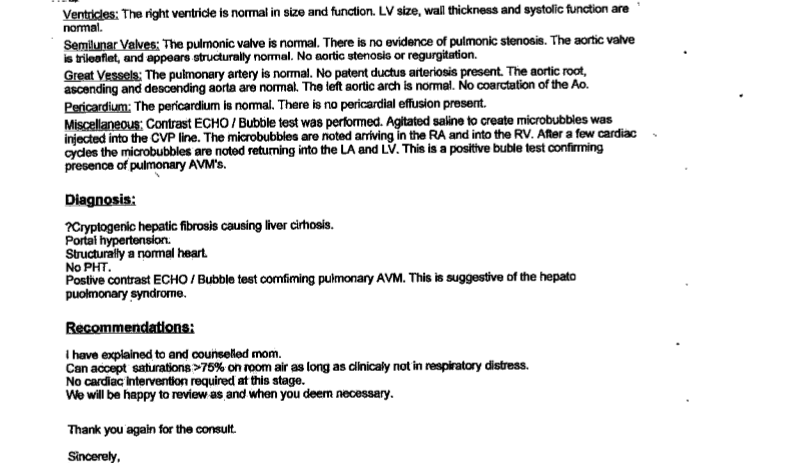

Supplement: Supplementary file 1 [file 7940365.f1.docx]
